# Health-Related Quality of Life among Egyptian Female Breast Cancer Patients at the National Cancer Institute, Cairo University

**DOI:** 10.31557/APJCP.2019.20.10.3113

**Published:** 2019

**Authors:** Amira I Khater, Maissa K Noaman, Marwa N Abdel Hafiz, Manar M Moneer, Inas A Elattar

**Affiliations:** *Department of Cancer Epidemiology and Biostatistics, National Cancer Institute, Cairo University, Egypt. *

**Keywords:** Quality of life, breast cancer, FACT, B+, Egypt

## Abstract

**Background:**

To measure the quality of life (QoL) of Egyptian females with breast cancer (BC) at the National Cancer Institute (NCI), Cairo University (CU) and its relations with the socio-demographic and clinical characteristics.

**Methods:**

A total of 200 female BC patients were recruited from the medical oncology outpatient clinic during a period from December 2015 to March 2018. The instrument of this study consisted of two parts: the first for Socio-demographic and clinicopathological characteristics, and the second was the Functional Assessment of Cancer Therapy-Breast for patients with Lymphedema (FACT-B+4) questionnaire.

**Results:**

The majority of the study participants were married, housewives, and without a family history of cancer (70.0%, 93.0%, and 63.0%, respectively). Most of them presented with breast mass, had IDC, grade II and disease stage III at diagnosis (89.0%, 84.5%, 85.6% and 56.8%, respectively) and had undergone modified radical mastectomy, received adjuvant chemotherapy, radiation, and hormonal therapy (62.0%, 83.8%, 73.5% and 60.5%, respectively). The median FACT-B score was 81 (range 35-133). The medians of subscales were: physical well-being 13 (range 0-28), social well-being 20 (range 0-28), emotional well-being 15 (range 2-24), and functional well-being 16 (range 2-28). The median score for breast subscale was 19 (range 2-32). Many factors affected the QoL scores, including age, marital status, occupation, smoking, residence, comorbidities, symptoms, grade, chemotherapy, radiation, and recurrence.

**Conclusion:**

QoL of Egyptian females with BC was influenced by several factors like age, marital status, occupation, smoking, residence, comorbidities, symptoms, grade, chemotherapy, radiation, and recurrence.

## Introduction

Breast cancer (BC) is the most commonly diagnosed cancer in women (24.2%). It is the most common in 154 of the 185 countries included in Globocan 2018. It is also the leading cause of cancer death in women (15.0%) (Bray et al., 2018). In the Middle East, BC represented 37.6%, 35.4%, 32.5%, 31.5% and 27.7% of all reported tumors in females in Egypt (1999-2001), Cyprus (1998-2001), Jordan (1996-2001), Israel-Jews (1996-2001) and Israel-Arabs (1996-2001), respectively (Freedman et al., 2006). According to the National Population-Based Cancer Registry Program in Egypt (2008-2011); BC is the most common cancer in females representing about 32% of all female malignancies with the crude and age-standardized incidence rates of 35.8 and 48.8 per 100,000 population, respectively (Ibrahim et al., 2014).

Improvement in early detection and treatment of BC has led to more prolonged survival of the patients. Breast cancer affects women’s identities and therefore studying the quality of life in women who lose their breasts is vital (Montazeri, 2008). Health-related quality of life refers to the extent to which one’s usual or expected physical, emotional, and social well-being are affected by a medical condition or its treatment (Cella and Nowinski, 2002). The time of diagnosis, initial stages of the treatment course, and the months following the end of treatment are hard times for patients both physically and emotionally. During these periods, poor adjustment and decreased quality of life in BC patients can easily occur (Hanson-Frost et al., 2000; Schnipper, 2001).

The aim of this study was to evaluate health-related quality of life (HRQoL) among the Egyptian female breast cancer patients at the National Cancer Institute (NCI), Cairo University (CU) and its relation with the demographic and clinicopathological characteristics.

## Materials and Methods

*Participants and procedures*

This hospital-based cross-sectional study included 200 Egyptian females with BC managed at NCI, CU. Participants were recruited from those attending for follow up at the medical oncology outpatient clinic during the period from December 2015 to March 2018. The study was approved by the Institutional Review Board at NCI, CU, and all participants signed informed consents. Eligible patients were asked to participate in this study after explaining its topic and objectives, and those who agreed were interviewed to collect the desired information. 

Inclusion criteria were adult females (>18 years old) with pathologically proved non-metastatic BC at diagnosis, following surgery with axillary lymph nodes dissection, at any stage of management either during chemotherapy, radiotherapy, hormonal or during their follow up after completing treatment and had no evidence of psychosis, dementia, or suicidal behavior. Pregnant females and those with cognitive or psychiatric illnesses were excluded. 

The data collection tool consisted of two parts; the first for socio-demographic and clinicopathological characteristics extracted after reviewing the patients’ medical records. The second part was the Functional Assessment of Cancer Therapy-Breast questionnaire (FACT-B+4). 

Socio-demographic characteristics included age, marital status, residence, occupation, education, and smoking status. Clinicopathological features were comorbidities, symptoms and signs, histopathological type, grade, stage, type of surgery, and other treatments (chemotherapy, radiotherapy or hormonal), and recurrence or metastasis if occurred. 

The FACT-B+4 questionnaire (Arabic version) consisted of 4 subscales: physical well-being (PWB), functional well-being (FWB), emotional well-being (EWB), social/family well-being (SWB) in addition to BC-specific concerns (BCS) and arm subscale. The investigator asked respondents to rate how true each statement was during the last seven days. It was measured on 5-point rating scales ranging from 0 (not at all) to 4 (very much). Negatively stated items were reversed by subtracting the response from 4. After reversing proper items, all subscale items were summed to a total, which is the subscale score. The higher the score the better the QOL. Kobeissi et al., (2014) translated, adapted, and face-validated FACT-B into Arabic. They concluded that it is an adequate instrument, appropriate for use, culturally sensitive, simple, and exhaustive. Coster et al., (2001) documented the validation of the arm subscale of FACT-B+4. The subscale showed good internal consistency (alpha coefficient = 0.62 to 0.88) and stability (test-retest reliability = 0.97).

*Sample size estimation*

Based on a previous study (Avis et al., 2005), the expected mean FACT-B score is 111, and standard deviation 19. A total sample size of 181 patients was needed to achieve a 95% confidence interval 5% around the assumed mean of 111. This sample was increased to 200 patients to allow for 10% nonresponse rate. Sample size was estimated using NQuery statistical package, version 7.0, Los Angeles, CA.

*Statistical analysis*

Data management and analysis were performed using Statistical Package for Social Sciences (SPSS) vs. 22. Numerical data were summarized using means and standard deviations for age and medians and ranges for the QoL scores. Categorical data were summarized as numbers and percentages. Comparison between two groups was done by Mann-Whitney test. Comparison between more than two groups was performed using the Kruskal-Wallis test. All tests were two-sided. A p-value < 0.05 was considered significant.

## Results

The mean age of the participants was 47.5±11.0 years (range: 26-80 years). The majority were married, housewives, and without a family history of cancer (70.0%, 93.0%, and 63.0%, respectively), Table 1.

Most of the participants presented with breast mass, had IDC, grade II and disease stage III at diagnosis (89.0%, 84.5%, 85.6%, and 56.8%, respectively) and had undergone MRM, received adjuvant chemotherapy, radiation and hormonal therapy (62.0%, 83.8%, 73.5%, and 60.5%, respectively), Table 2.

**Table 1:** Demographic and Personal Characteristics

Characteristics	Number n=200	Percent (%)
Age at diagnosis		
<50 years	117	58.5
≥50 years	83	41.5
Marital status		
Married	140	70
Not married	60	30
Residence		
Greater Cairo	152	76
Outside Greater Cairo	48	24
Education		
Not Educated	84	42
Primary& Preparatory	40	20
Secondary & College and above	76	38
Occupation		
Working	14	7
House wife	186	93
Family history of cancer		
No	126	63
Yes	74	37
Smoking status		
Non smokers	127	63.5
Passive smokers	73	36.5
Comorbidities		
No	112	56
Yes	88	44

**Table 2:** Clinical and Management Characteristics

Characteristics	Number n=200	Percent (%)
Symptoms and Signs		
Pain	41	20.5
Other Symptoms	159	79.5
Histopathology		
Invasive duct carcinoma	169	84.5
Other types	31	15.5
Grade (n=181)		
Grade 2	155	85.6
Grade 3	26	14.4
Stage at diagnosis (n=169)		
Stage I & II	73	43.2
Stage III	96	56.8
Type of surgery		
Radical and modified radical mastectomy	125	62.5
Conservative + lymph node resection and simple mastectomy	75	37.5
Chemotherapy (n=198)*		
Adjuvant	166	83.8
Neoadjuvant and Adjuvant	32	16.2
Radiation therapy	147	73.5
Hormonal therapy	121	60.5
Recurrence	44	22
Reconstruction	6	3
Post-surgery infection	9	4.5

* Two patients did not receive any type of chemotherapy

**Table 3:** Quality of Life Scores

Quality of life characteristics	Median (range)
FACT-B total score	81 (35-133)
Subscale	
Social well-being	20 (0-28)
Breast cancer	19 (2-32)
Functional well-being	16 (2-28)
Emotional well-being	15 (2-24)
Physical well-being	13 (0-28)
Arm	10 (0-20)

**Table 4:** Quality of Life in Relation to the Demographic Characteristics

Characteristics	N=200	PWB	SWB	EWB	FWB	BCS	Arm	TQOL
Age at diagnosis								
<50 years	117	13 (1-28)	20 (0-28)	15 (2-24)	17 (2-28)	17 (2-31.5)	10 (1-20)	80 (35-133)
≥50 years	83	11 (0-23)	21 (7-28)	15 (4-24)	15 (3-26)	20.3 (4.5-32.0)	10 (0-20)	82 (44-126)
P-value		0.085	0.517	0.883	0.012	0.044	0.452	0.676
Marital status								
Married	140	13 (0-28)	20 (7-28)	15 (2-24)	16 (2-28)	18.5 (2-32)	9.5 (0-20)	82 (35-133)
Not married	60	12.5 (1-28)	20.4 (0-28)	13.5 (4-24)	15 (3-28)	19.1 (4.5-31.5)	10.5 (0-20)	80.8 (39-128)
P-value		0.293	0.759	0.077	0.021	0.359	0.312	0.241
Residence								
Greater Cairo	152	13 (0-28)	20 (7-28)	14 (2-24)	16 (2-28)	18.5 (2-32)	10 (0-20)	81 (35-126)
Outside Greater Cairo	48	13 (4-28)	21.5 (0-28)	15 (6-24)	16 (8-28)	21 (10-31.5)	10 (1-20)	87 (39-133)
P-value		0.181	0.040	0.052	0.096	0.025	0.439	0.025
Education								
Not educated	84	11 (1-28)	19.8 (9-28)	14 (2-24)	15 (2-28)	19 (3- 31.5)	9 (0-20)	78 (35-128)
Primary & Preparatory	40	12.5 (0-25)	21.5 (11-28)	15 (4-23)	16.5 (6-28)	21.2 (9-32)	10 (0-18)	87 (45-126)
Secondary & College and above	76	14 (1-26)	20 (0-28)	15 (4-24)	17.5 (3-28)	18 (2-29)	10 (1-20)	84 (39-133)
P-value		0.214	0.096	0.194	0.052	0.505	0.782	0.155

PWB, Physical well-being; SWB, Social well-being; EWB, Emotional well-being; FWB, Functional well-being; BCS, Breast cancer subscales; Arm, Arm subscale; TQOL, Total quality of life; Data were presented as median (range).

**Table 5:** Quality of Life in Relation to the Personal Characteristics

Characteristics	N=200	PWB	SWB	EWB	FWB	BCS	Arm	TQOL
Occupation								
Working	14	14 (11-20)	20 (7-28)	16.5 (9-20)	15.5 (8-21)	19 (14-25)	10.5 (1-17)	88 (67-104)
House wife	186	12.5 (0-28)	20 (0-28)	15 (2-24)	16 (2-28)	19 (2-32)	10 (0-20)	80 (35-133)
P-value		0.024	0.683	0.202	0.752	0.479	0.628	0.367
Family history of cancer								
No	126	13 (1-28)	20 (7-28)	15 (2-24)	17 (2-28)	19 (2-32)	10 (0-20)	82 (35-133)
Yes	74	12.5 (0-26)	20 (0-28)	15 (4-24)	15.5 (3-27)	19 (4-29)	10 (0-20)	81 (39-126)
P-value		0.106	0.841	0.918	0.174	0.599	0.694	0.307
Smoking status								
Non smokers	127	13 (0-28)	21 (0-28)	14 (4-24)	16 (3-28)	18 (2-31.5)	10 (0-20)	81 (39-133)
Passive smokers	73	14 (1-28)	19 (7-28)	15 (2-24)	16 (2-28)	19 (3-32)	11 (1-20)	84 (35-126)
P-value		0.326	0.016	0.153	0.942	0.094	0.013	0.543
Comorbidities								
No	112	13 (1-28)	21 (9-28)	15 (4-24)	16.5 (3-28)	18 (3-32)	10.5 (0-20)	84 (40-133)
Yes	88	12 (0-25)	19.4 (0-28)	15 (2-24)	15 (2-27)	19 (2-29.3)	9 (0-20)	80 (35-120)
P-value		0.181	0.014	0.625	0.004	0.976	0.444	0.057

PWB, Physical well-being; SWB, Social well-being; EWB, Emotional well-being; FWB, Functional well-being; BCS, Breast cancer subscales; Arm, Arm subscale,; TQOL, Total quality of life; Data were presented as median (range).

**Table 6:** Quality of Life in Relation to the Clinicopathological Characteristics (n=200)

Characteristics	n	PWB	SWB	EWB	FWB	BCS	Arm	TQOL
Symptoms & Signs								
Pain	41	11 (1-22)	19 (7-28)	13 (4-24)	15 (3-25)	18 (3-29)	10 (0-20)	77 (40-112)
Other Symptoms	159	13 (0-28)	21 (0-28)	15 (2-24)	17 (2-28)	19 (2-32)	10 (0-20)	83 (35-133)
P-value		0.067	0.354	0.081	0.036	0.274	0.414	0.036
Histopathology								
Invasive duct carcinoma	169	13 (0-28)	20 (7-28)	15 (2-24)	16 (2-28)	19 (2-32)	10 (0-20)	81 (35-128)
Other types	31	9 (1-26)	20 (0-28)	15 (4-24)	14 (3-28)	19 (7-29)	10 (0-20)	84 (39-133)
P-value		0.089	0.638	0.990	0.136	0.907	0.894	0.310
Grade (n=181)								
Grade 2	155	13 (0-28)	20 (7-28)	15 (4-24)	16 (3-28)	19 (2-29)	10 (0-20)	81 (40-133)
Grade 3	26	14 (2-28)	22 (15-28)	15.5 (5-24)	18.5 (8-28)	22.7 (10-32)	11 (1-20)	88 (59-128)
P-value		0.210	0.022	0.047	0.091	0.013	0.186	0.014
Stage (n=169)								
Stage I & II	73	13 (1-26)	20 (0-28)	15 (4-24)	16 (3-28)	19 (7-29.3)	11 (1-20)	83 (39-133)
Stage III	96	14 (0-28)	21 (7-28)	15 (4-24)	17 (3-28)	19 (3-32)	9 (0-20)	83 (40-128)
P-value		0.655	0.467	0.758	0.568	0.123	0.116	0.978

PWB, Physical well-being; SWB, Social well-being; EWB, Emotional well-being; FWB, Functional well-being; BCS, Breast cancer subscales; Arm, Arm subscale; TQOL, Total quality of life; Data were presented as median (range).

**Table 7:** Quality of Life in Relation to Management Characteristics

Characteristics	n=200	PWB	SWB	EWB	FWB	BCS	Arm	TQOL
Type of surgery								
Radical and modified radical mastectomy	125	13 (0-28)	21 (0-28)	15 (2-24)	16 (2-28)	19 (2-32)	10 (0-20)	81 (35-133)
Conservative + lymph node resection and simple mastectomy	75	14 (2-26)	20 (9-28)	15 (4-23)	16 (3-28)	19 (7-29)	11 (0-20)	83 (40-118)
P-value		0.527	0.473	0.806	0.847	0.500	0.345	0.755
Chemotherapy (n=198)								
Adjuvant Only	166	13 (0-28)	21 (0-28)	15 (4-24)	16 (3-28)	19 (3-32)	10.5 (0-20)	84 (39-133)
Neoadjuvant + Adjuvant	32	10 (1-22)	18 (7-28)	12 (2-24)	15 (2-25)	17 (2-25)	6 (1-20)	70 (35-112)
P-value		0.027	0.006	0.006	0.150	0.006	0.004	0.001
Radiation therapy								
No	53	13 (1-28)	21 (7-28)	16 (4-24)	16 (6-28)	19 (2-28)	10 (0-20)	86 (40-133)
Yes	147	13 (0-28)	20 (0-28)	14 (2-24)	16 (2-28)	19 (4-32)	10 (0-20)	81 (35-128)
P-value		0.844	0.400	0.044	0.301	0.689	0.711	0.764
Hormonal therapy								
No	79	13 (0-28)	21 (7-28)	15 (4-24)	16 (3-28)	18 (2-29)	10 (0-20)	84 (40-133)
Yes	121	13 (1-28)	20 (0-28)	14 (2-24)	16 (2-28)	19 (4-32)	10 (0-20)	80 (35-128)
P-value		0.834	0.183	0.065	0.681	0.302	0.986	0.673
Recurrence								
No	156	13 (0-28)	21 (0-28)	15 (2-24)	16 (2-28)	19 (3-32)	10 (0-20)	83 (35-133)
Yes	44	13 (1-28)	18.7 (7-28)	12 (4-24)	15.5 (6-27)	17.5 (2-28.1)	10 (0-18)	73 (40-119)
P-value		0.793	0.013	0.011	0.097	0.135	0.501	0.022
Reconstruction								
No	194	13 (0-28)	20 (0-28)	15 (2-24)	16 (2-28)	19 (2-32)	10 (0-20)	81 (35-133)
Yes	6	13.5 (7-19)	19.4 (18-28)	16.5 (13-19)	20 (15-25)	19.2 (14-28)	10.5 (2-15)	88 (80-111)
P-value		0.638	0.709	0.300	0.103	0.557	0.593	0.196
Post-surgery infection								
No	191	13 (0-28)	20 (0-28)	15 (2-24)	16 (2-28)	19 (2-32)	10 (0-20)	81 (35-133)
Yes	9	13 (5-18)	19.8 (9-26)	15 (9-23)	17 (8-23)	18 (9-24)	9 (1-18)	75 (57-100)
P-value		0.92	0.962	0.802	0.904	0.697	0.280	0.775

PWB, Physical well-being; SWB, Social well-being; EWB, Emotional well-being; FWB, Functional well-being; BCS, Breast cancer subscales; Arm, Arm subscale; TQOL, Total quality of life; Data were presented as median (range).

FACT-B+4 total questionnaire had a median score of 81 (range 35-133); subscales are shown in Table 3. Functional well-being score was significantly higher in the younger age group (p=0.012) and married patients (p=0.021). A significantly higher score of BC subscale was observed in the older age group (p=0.044). Those who reside outside greater Cairo had significantly higher scores of SWB, BC subscales, and the total quality of life score than greater Cairo residents (p=0.040, p=0.025 and p= 0.025, respectively), Table 4.

Working patients had significantly higher PWB score than housewives (p=0.024). Passive smokers had significantly lower SWB score (p=0.016) and significantly higher arm subscale score than non-smokers (p=0.013). Patients who had no comorbidities had significantly higher SWB and FWB scores than those who had comorbidities (p=0.014 and p=0.004 respectively), Table 5.

Patients who had symptoms other than pain had higher FWB and the total quality of life scores than those who had pain (p=0.036, p=0.036, respectively). Participants with grade 3 had significantly higher SWB, EWB, BC subscale, and the total quality of life scores than those with grade 2 (p=0.022, p=0.047, p=0.013, and p=0.014, respectively), Table 6.

Patients who received adjuvant chemotherapy had significantly higher PWB, SWB, EWB, BC, arm subscales and total quality of life scores than those who received neoadjuvant and adjuvant chemotherapy (p= 0.027, p=0.006, p=0.006, p=0.006, p=0.004, and p=0.001, respectively), Table 7.

Patients who hadn’t received radiation therapy had significantly higher EWB score than those who received that therapy (p=0.044). Patients who didn’t develop recurrence had higher SWB, EWB, and total quality of life scores than those who developed a recurrence (p=0.013, p=0.011, and p=0.022, respectively), Table 7.

## Discussion

This study demonstrated that QoL of Egyptian females with BC was influenced by many factors including age, marital status, occupation, smoking, residence, comorbidities, symptoms, grade, chemotherapy, radiation, and recurrence.

The current study showed a significantly higher score of BC subscale in the older age group (≥ 50 years old) compared to the younger age group (< 50 years old), p=0.044. This agreed with what concluded from another study conducted in the Levant by Akel et al., (2017). On the other hand, the younger age group had a significantly higher FWB score (p=0.012) than the older group; this could be explained by the fact that younger woman may have young children who need more service and care and may have more ambitions to achieve. Functional well-being score was also higher in married patients (p=0.021). Married women may have more home responsibilities regarding caring for their children and husbands, which make them do more work.

Those who reside outside greater Cairo had significantly higher scores of SWB, breast cancer subscales, and the total quality of life score than greater Cairo residents (p=0.040, p=0.025 and p= 0.025 respectively). Family and neighborhood relationships tend to be stronger and closer among those residing outside greater Cairo. When a woman gets sick, her relatives and neighbors reassure, encourage her to take and complete her treatment and give her support in different ways; being beside her especially after surgery and doses of chemotherapy, serving her and complete her domestic work if she cannot do that and take care of her children. These relationships and support are weak and almost non-existent among those residing in the Greater Cairo area.

Working patients had significantly higher PWB score than housewives (p=0.024). Working women do physical activity to cope with their work besides the usual household duties. But, this difference was not found in a study conducted by Al-Naggar et al., (2011).

Patients who had no comorbidities had significantly higher SWB and FWB scores than those who had comorbidities (p=0.014 and p=0.004, respectively). Irukulla et al., (2016) concluded that comorbidities negatively affected multiple QoL domains. They found that patients with diabetes and hypertension had significantly lower scores in physical functioning in comparison to patients without these diseases. 

Pain was associated with lower FWB and the total quality of life scores compared to other symptoms (p=0.036, for both). Costa et al., (2017) conducted a study to evaluate the influence of pain on QoL in BC patients and concluded that pain compromises the QoL of BC patients, particularly those with advanced stages of the disease.

Patients who did not receive radiation therapy had significantly higher EWB score than those who received that therapy (p=0.044). This agrees partially with that concluded by Al-Naggar et al., (2011), who reported a negative impact of radiotherapy on EWB and FWB. A Chinese study reported lower scores in physical and social domains and overall QOL in patients who received radiotherapy (Lu et al., 2007). Another study in Iraq found a significant negative association between radiotherapy and PWB (Al-Naggar et al., 2016). In contrast, Cui et al., (2004) found no association between QoL and radiotherapy. 

Diagnosis of recurrent cancer is a significant stressor that can worsen the QoL (Okamura et al., 2005). This comes in agreement with our findings where SWB, EWB, and total QoL scores were negatively affected by the occurrence of recurrence. Hamer et al., (2017) determined the QoL and symptom burden among duct carcinoma in situ (DCIS), early stage, locally advanced and metastatic breast cancer patients using the FACT-B questionnaire. They concluded that patients with metastatic disease had the lowest QoL in all of the five domains and total QoL compared to all other cancer stages. Metastatic patients had greater pain, drowsiness, dyspnea, and appetite loss compared to all other patient groups. They also had more depression, fatigue, and anxiety compared to DCIS and early-stage patients.

The results of this study revealed that the median FACT-B score was 81 (range 35-133). Medians for various subscales scores were: PWB 13 (range 0-28), SWB 20 (range 0-28), EWB 15 (range 2-24), and FWB 16 (range 2-28). The median score for breast subscale was 19 (range 2-32). All these scores were slightly lower than those reported by Lopes et al., (2018) who assessed the QoL in Brazilian women with BC. They found that the mean FACT-B score was 85.8±18.1. Mean score for various subscales were: PWB 22.2±7.8, SWB 21.5±5.7, EWB 19.7±4.6 and FWB 22.3±5.4. The mean score for breast subscale was 24.4±7.9.

Another Japanese study reported obviously higher scores compared to the current study. Ohsumi et al., (2009) compared QoL between patients who had a mastectomy and those who had breast-conserving treatment (BCT). They reported a mean FACT-B score of 115.4±14.5 and 118.2±15.6 in the two groups, respectively, with no significant difference between the two groups. The mean score for various subscales were: PWB 26.5±2.1 and 26.1±2.9, SWB 21.1±8.7 and 24.0±10.5, EWB 20.0±3.0 and 20.0±3.4 and FWB 22.1±5.2 and 23.3±5.1 for BCT and mastectomy groups, respectively. The mean score for breast subscale was 25.6±4.4 for BCT group and 24.0 ± 4.4 for mastectomy group (Ohsumi et al., 2009). Likewise, in the current study, the type of surgery had no significant effect on the total FACT-B score or any of its subscales.

The following points may explain lower scores in the current study compared to the last two studies. In the first study, they included only breast cancer women who had completed their treatment and had been receiving clinical follow-up care for at least 12 months from the date of the last therapeutic procedure and also in the second study, they included breast cancer patients who had at least 5 years follow up after their definitive surgeries. On the other hand, 92% of our study participants were still receiving their treatments (chemo, radio, or hormonal therapy) when interviewed.

Breast cancer patients receiving chemotherapy might experience several side-effects and symptoms that have a negative effect on their quality of life. Also, adjuvant hormonal therapies were found to have a similar negative impact on the quality of life (Paraskevi, 2012). In the current study, neoadjuvant therapy had a negative impact on QoL compared to patients who received only adjuvant chemotherapy.

A study by Beaulac et al., (2002) was conducted to detect QoL among BC patients who developed lymphedema and those who didn’t in Massachusetts, United States. They reported a mean FACT-B score of 109.1±2.9 and 122.7±1.4 for the two groups, respectively. The mean score for various subscales were: PWB 23.1±0.7 and 26.0±0.3, SWB 23.7±1.0 and 24.6±0.5, EWB 18.8±0.7 and 20.6±0.3 and FWB 21.2±0.8 and 24.4±0.4 for lymphedema and no lymphedema groups, respectively. The mean score for breast subscale was 22.4±1.1 for lymphedema group and 27.2±0.5 for the non-lymphedema group. All these scores are higher than those in the current study which may be because their study included only early stages of the disease (ductal carcinoma in situ, stages I, II) while the present study included stages I, II, III.

The small number of patients in some of the subgroups and few missed data of grade and stage of some patients are the limitations of this study. To overcome the problem of small numbers, we resorted to combining the small numbers to other groups for better analytical results. In conclusion, QoL of Egyptian females with BC at the National Cancer Institute was influenced by many factors including age, marital status, occupation, smoking, residence, comorbidities, symptoms, grade, chemotherapy, radiation, and recurrence.

## References

[B1] Akel R, El Darsa H, Anouti B (2017). Anxiety, depression and quality of life in breast cancer patients in the levant. Asian Pac J Cancer Prev.

[B2] Al-Naggar R, Osman M, Al-Baghdadi N (2016). Study of quality of life and characteristic factors in women with breast cancer undergoing different types of therapy. J Appl Pharm Sci.

[B3] Al-Naggar RA, Nagi NM, Ali MM, Almuasli M (2011). Quality of life among breast cancer patients in Yemen. Asian Pac J Cancer Prev.

[B4] Avis NE, Crawford S, Manuel J (2005). Quality of life among younger women with breast cancer. J Clin Oncol.

[B5] Beaulac SM, McNair LA, Scott TE, LaMorte WW, Kavanah MT (2002). Lymphedema and quality of life in survivors of early-stage breast cancer. Arch Surg.

[B6] Bray F, Ferlay J, Soerjomataram I (2018). Global cancer statistics 2018: GLOBOCAN estimates of incidence and mortality worldwide for 36 cancers in 185 countries. CA Cancer J Clin.

[B7] Cella D, Nowinski CJ (2002). Measuring quality of life in chronic illness: the functional assessment of chronic illness therapy measurement system. Arch Phys Med Rehabil.

[B8] Costa WA, Monteiro MN, Queiroz JF, Gonçalves AK (2017). Pain and quality of life in breast cancer patients. Clin Sao Paulo Braz.

[B9] Coster S, Poole K, Fallowfield LJ (2001). The validation of a quality of life scale to assess the impact of arm morbidity in breast cancer patients post-operatively. Breast Cancer Res Treat.

[B10] Cui Y, Shu X-O, Gao Y (2004). The long-term impact of medical and socio-demographic factors on the quality of life of breast cancer survivors among Chinese women. Breast Cancer Res Treat.

[B12] Hamer J, McDonald R, Zhang L (2017). Quality of life (QOL) and symptom burden (SB) in patients with breast cancer. Support Care Cancer.

[B13] Hanson Frost M, Suman VJ, Rummans TA (2000). Physical, psychological and social well-being of women with breast cancer: the influence of disease phase. Psychooncology.

[B14] Ibrahim AS, Khaled HM, Mikhail NN, Baraka H, Kamel H (2014). Cancer incidence in egypt: results of the national population-based cancer registry program. J Cancer Epidemiol.

[B15] Irukulla M, Vaghmare R, Joseph D (2016). Impact of comorbidities on quality of life in breast cancer patients. Indian J Cardiovasc Dis Women.

[B16] Kobeissi L, Saad MA, Doumit M (2014). Face validity of the functional assessment of cancer therapy-breast symptom index (FACT- B) into formal Arabic. Middle East J Cancer.

[B17] Lopes JV, Bergerot CD, Barbosa LR (2018). Impacto del cáncer de mama y calidad de vida de mujeres sobrevivientes. Rev Bras Enferm.

[B18] Lu W, Cui Y, Zheng Y (2007). Impact of newly diagnosed breast cancer on quality of life among Chinese women. Breast Cancer Res Treat.

[B19] Montazeri A (2008). Health-related quality of life in breast cancer patients: a bibliographic review of the literature from 1974 to 2007. J Exp Clin Cancer Res.

[B20] Ohsumi S, Shimozuma K, Morita S (2009). Factors associated with health-related quality-of-life in breast cancer survivors: Influence of the type of surgery. Jpn J Clin Oncol.

[B21] Okamura M, Yamawaki S, Akechi T, Taniguchi K, Uchitomi Y (2005). Psychiatric disorders following first breast cancer recurrence: prevalence, associated factors and relationship to quality of life. Jpn J Clin Oncol.

[B22] Paraskevi T (2012). Quality of life outcomes in patients with breast cancer. Oncol Rev.

[B23] Schnipper HH (2001). Life after breast cancer. J Clin Oncol.

